# Mandible microwear texture analysis of crickets raised on diets of different abrasiveness reveals universality of diet-induced wear

**DOI:** 10.1098/rsfs.2023.0065

**Published:** 2024-04-12

**Authors:** Daniela E. Winkler, Hitomi Seike, Shinji Nagata, Mugino O. Kubo

**Affiliations:** ^1^ Kiel University, Zoological Institute, Zoology and Functional Morphology of Vertebrates, Kiel, Schleswig-Holstein, Germany; ^2^ Department of Natural Environmental Studies, The University of Tokyo, Graduate School of Frontier Sciences, Kashiwa, Chiba, Japan; ^3^ Department of Integrated Biosciences, The University of Tokyo, Graduate School of Frontier Sciences, Kashiwa, Chiba, Japan

**Keywords:** microwear, diet reconstruction, dental wear, tribology

## Abstract

Animals have evolved diverse comminuting tools. While vertebrates possess mineralized teeth, insect mandibles often bear metal-inclusion-hardened serrated cusps. Microscopic dental enamel wear (microwear) is known to be caused by contact with ingesta. To test if insect mandible microwear is also diet-dependent, we kept newly moulted adult two-spotted crickets (*Gryllus bimaculatus*) for four weeks on alfalfa-based rodent pellets with and without added mineral abrasives (loess, quartz, volcanic ash). Six crickets per diet were examined after 1, 3, 7, 14, 21 and 28 days. All diets induced progressive mandible wear, affecting specific locations along the distal tooth cusps differently. The depth of furrows increased on most abrasive-containing diets until day 21, while wear mark complexity increased from day 1 to 3 and 14 to 21. After 28 days, these parameter values for large volcanic ash and large quartz diets significantly exceeded those for the control diet. These results are comparable to observations from guinea pig feeding experiments with the same diets. Cricket mandible wear was affected by all abrasives. Notably, large volcanic ash and large quartz induced the deepest, most complex lesions, akin to observations in guinea pigs. This suggests a universal wear process, supporting that microwear analyses are suitable for inferring invertebrate diets.

## 1. Introduction

Efficient breakdown of plant material for digestion is a critical process among herbivorous vertebrates and invertebrates, facilitated by specialized cutting tools such as teeth or toothed mandibles [1], both fortified by inorganic components. While vertebrates predominantly rely on calcium and phosphate, forming hydroxyapatite in teeth, invertebrates demonstrate the incorporation of a diverse array of metals like zinc, manganese, iron and calcium into their mandibles [2]. Plants have evolved multifaceted defence mechanisms against herbivory [3], ranging from visible morphological defence structures, such as thorns and trichomes [4,5] or the production of secondary metabolites which act as deterrents against herbivores [6]. But also on a cellular level, the amorphous silica inclusions often embedded in the plant tissue, the so-called phytoliths, are considered a natural deterrent against herbivores, both vertebrate and invertebrate [7,8]. Studies on mammalian herbivores have elucidated the role of phytoliths in dental wear through controlled feeding experiments [9–12] and through comparisons of wild populations with different food habits [13–15]. Moreover, evidence in invertebrates suggests a significant impact of phytoliths on mandible wear. For instance, silica fertilizer treatments have been linked to increased mandible wear in the African stalk borer [16] and the sugarcane early shoot borer [17], proposing silica as a potential invertebrate deterrent for crops. Further substantiating this link, investigations on the African armyworm revealed differences in mandible wear between phytolith-rich diets and those with lower silica content, highlighting the influence of diet on wear patterns in invertebrates [18]. The link between diet and mandible wear, also under natural feeding regimes, and over an insect's lifespan, has been established in many other species including leaf beetle [19], true bugs [20], stink bug [21], leaf cutter ants [22], and spiny leaf insect irrespective of diet [23]. Existing methodologies predominantly rely on absolute mandible tooth length and angle measurements [16,18,21] or digital image analysis of worn areas [24] to quantify wear, limiting cross-species comparability. Consequently, a standardized, repeatable, and transferrable approach to quantify mandible wear and investigate potential diet-specific wear patterns induced by different abrasiveness of the feed would greatly improve utilization of mandible wear as a proxy for inferring diet and reconstructing habitats among invertebrates, offering novel insights into their ecological niches and behaviours.

To facilitate this approach, the following basic questions need to be addressed:

1. How long does it take until visible wear marks form on insect mandibles on specific diets?
2. Does the wear progress over the course of an insect's lifespan?
3. Can insect mandible wear be objectively quantified?
4. Is insect mandible wear diet-specific?

To address these four questions, we performed a controlled feeding experiment using a staggered-killing design with the two-spotted cricket (*Gryllus bimaculatus*). The two-spotted cricket usually has eight nymphal instars followed by the adult stage, in which it reproduces. In crickets, number of instars can vary with temperature [25,26]. Adult crickets live between four and six weeks [27,28] and continuously consume food during their active reproduction. They are therefore an ideal model to observe mandible wear. Crickets were reared on standardized pelleted diets based on alfalfa, to ensure that diets did not contain silica from phytoliths. The base-pellet was then reprocessed and selected mineral abrasives of different size, quantity and geometry were added to the pellet. Thus, diets consisting of a comparable base matrix but of different inherent abrasiveness were created. To quantify mandible wear, we adapted dental microwear texture analysis (DMTA), a method originally developed for mammals and extended to diverse non-mammalian species, including lepidosaurs [29,30], bony fish [31], sharks [32,33], alligators [34] and dinosaurs [35–37]. DMTA quantifies microscopic wear patterns (topography) of enamel wear facets. Controlled feeding experiments have established that the observed wear patterns depend on physical characteristics of the diet, such as phytolith [11] and water content [12], hardness [34,38], and can be affected by additional ingesta such as grit and mineral abrasives [39,40]. For this experiment, the effect of mineral abrasives on cricket mandible wear will be in focus, using experimental diets which differ in abrasiveness.

## 2. Material and methods

### 2.1. Animals, rearing and diets

**Figure 1.**
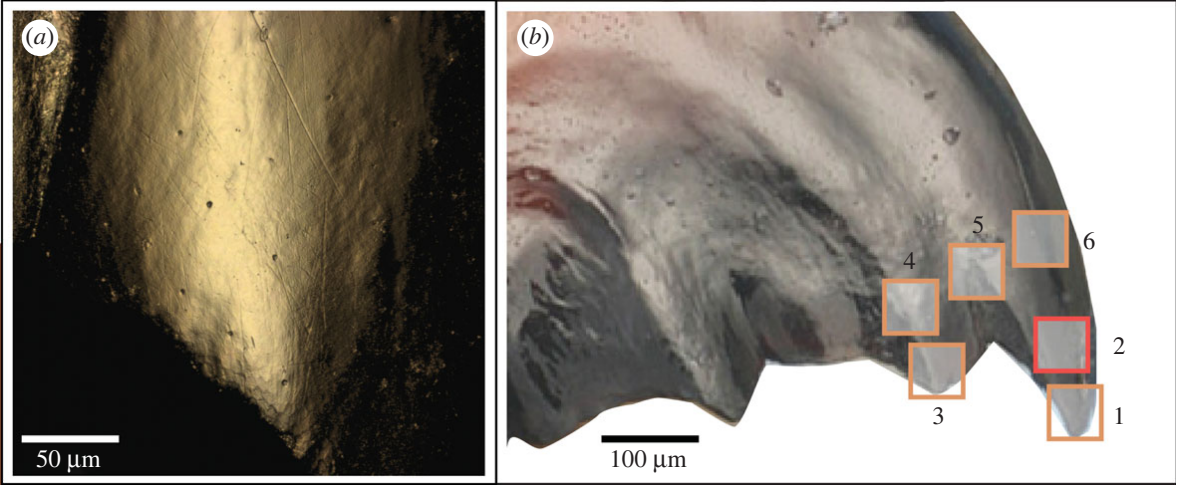
Overview of mandible morphology. (*a*) Individual kept without food for one day after moulting. Only very scarce and shallow wear marks are visible on the mandible. (*b*) Approximate positioning of the six measurement locations on the first and second anteriormost tooth of the external side of the left mandible. Location 2 was selected as a focal scanning location for further analyses.

Newly moulted adult two-spotted crickets (*Gryllus bimaculatus*) were obtained from the Nagata laboratory at the University of Tokyo, where they are continuously bred and reared for other studies. As the crickets were isolated from their breeding facility during final instar moulting or immediately after moulting, while their exoskeletal cuticle was still soft, it is unlikely that they had already ingested food with their newly moulted mandibles. Thus, mandibles were expected to be unworn in these individuals. We visually confirmed the wear state of mandibles in six individuals that were kept for one day without food after adult emergence (figure 1*a*) and included these specimens into further analysis. Crickets were reared in groups of three in insect boxes (w10 cm × d15 cm × h10 cm) and provided with moist paper tissue as a water source and ad libitum food. Boxes were enriched with dry paper towels to provide shelter and allow the individuals to spatially separate. The room was airconditioned (25°C) and on a 16 h/8 h day and night cycle.

We included six different experimental diets, of which five were used in a previous feeding experiment with guinea pigs [40]. As the abrasive-free control diet used in [40] was not available, we replaced it with a different alfalfa-based pellet (all diet formulas are included in the appendix, see electronic supplementary material, table S1). The pelleted diets included a variety of mineral abrasives of different type, quantity and geometry. We chose 4% loess (4Ls), 8% silt-sized quartz (8sS), 4% small volcanic ash (4sVA), 4% large volcanic ash (4lVA) and 4% fine sand-sized quartz (4lS). The mean diameter and form factor of abrasive particles were determined in [40] and are given in table 1.

**Table 1.** Characteristics of the abrasives included into the 6 experimental pelleted diets.

diet	abbreviation and colour code	primary mineral phase	abrasive concentration (%)	mean size^a^ ± s.d. (μm)	mean size^b^ (μm)	form factor^b^
control	C (blue)	—	0	—	—	—
small quartz	8sS (yellow)	quartz	8	4.41 ± 1.39	5.90	0.36
loess	4Ls (green)	quartz	4	36.87 ± 3.57	31.00	0.55
small volcanic ash	4sVA (orange)	amorphous silica	4	24.19 ± 2.48	20.34	0.63
large volcanic ash	4lVA (pink)	amorphous silica	4	96.44 ± 9.03	64.57	0.49
large quartz	4lS (red)	quartz	4	166.01 ± 15.47	143.83	0.77

^a^Data from laser diffraction size analysis.

^b^Data from QUEMSCAN.

Six crickets each were maintained solely on their designated experimental diet and water for 1, 3, 7, 14, 21, and 28 days, resulting in a total of *n* = 216 individuals. Six additional individuals were kept for one day without food as controls for unworn mandibles. All individuals (*n* = 222) were euthanized by freezing them at −18°C for at least 24 h.

We confirmed that the crickets ingested abrasives with the diet by performing X-ray diffraction (XRD) analysis on hand-milled faecal samples on a ‘D8 Discover’ X-ray diffractometer by Bruker AXS (using a theta–theta geometry and equipped with a copper X-ray source) at the Petrology laboratory, Institute of Geosciences, Kiel University. Faecal samples of crickets were collected for 7 days during the experiment, air-dried and then frozen at −18°C. In order to create a calibration dataset for quartz content, aliquots of 300–600 mg of quartz-free rabbit feed were additionally mixed with 0, 1, 2, 5 and 10 wt% of ultrapure quartz meal to determine the height of the quartz peak associated with these quartz contents [12,40]. Results of XRD analyses are included in the electronic supplementary material (figure S1).

### 2.2. Preparation and data acquisition

For each individual, the left mandible was carefully dissected under a stereomicroscope. Isolated mandibles were then cleaned using the following protocol: submerging them for 5 min in an ultrasonic bath using a 2.5% NaClO solution, followed by 5 min in an ultrasonic bath using Milli-Q water, and finally swiping them with cotton swaps soaked in acetone. If dirt particles or the top wax layer of the exoskeletal cuticle were still visible under 20× magnification, mandibles were swiped with cotton swaps soaked in *n*-hexane. For the groups kept on 4% large volcanic ash (4lVA) and 4% small volcanic ash (4sVA), one individual each from day 1, and for 4lVA from day 7, could not be included into further analysis due to substantial damages to the mandibles during preparation.

**Figure 2.**
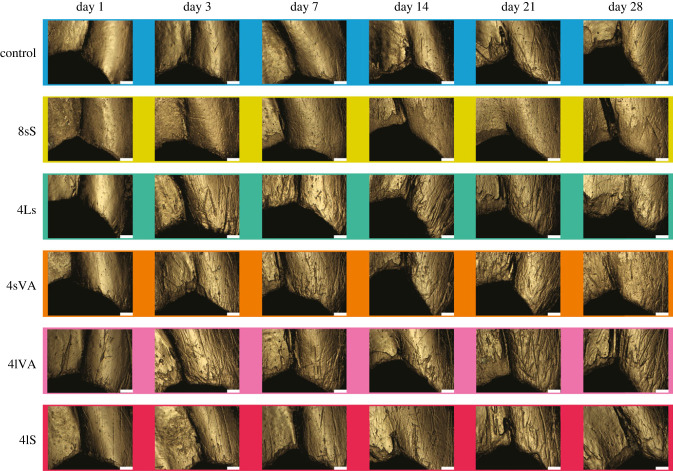
Progressive mandible wear on the two anteriormost mandible teeth of crickets raised on diets of different abrasiveness over the course of the experiment. The left mandible from external view of one random individual per day and diet is shown. Scale bar, 100 µm.

We then first acquired overview images with a 20× long working distance lens (figure 2) and then subsequently topographical surface scans using a confocal laser-scanning microscope (VK-9700, Keyence, Osaka, Japan) at the Kubo laboratory (University of Tokyo), with a violet laser (408 nm), equipped with a long working distance 100× lens (NA = 0.73) (resolution in *x*, *y* = 0.138 µm, step size in *z* = 0.001 µm). Scans were obtained from the external side of the mandible. For each specimen, six surface scans of comparable location (scan size 102 µm × 120 µm) along the first and second mandibular tooth were taken (figure 1*b*). Each surface scan was trimmed to 60 µm × 60 µm using MountainsMap v. 9.0.9878 (DigitalSurf, Besançon, France). Visual inspection of the scans showed that scanning locations 1, 3 and 5 were often worn to an extreme degree, resulting in broken tips and very deep scars (figure 2). We therefore discarded these locations and only focused on the scanning locations 2, 4 and 6. DMTA was conducted in MountainsMap, applying the published filtering routine for data obtained on the VK-9700 [15,41], which includes the following steps: levelling (least-square plane by subtraction), spatial filtering (robust Gaussian filter with a cut-off value of 0.8 µm), filling of non-measured points using the smoothing function of MountainsMap, noise reduction by thresholding (upper and lower 0.5%), removal of outliers (maximum slope of 85%) and form removal (polynomial of increasing power = 2). Forty-two DMTA parameters were calculated (for parameter descriptions, see electronic supplementary material, table S2). Boxplots of all 42 DMTA parameters are included in the electronic supplementary material (figure S2). We do not include direction-related parameters that represent absolute orientation (in degrees) of wear features, as they likely represent movement of the mandible against the diet during processing and are not expected to indicate dietary differences. Because of the large number of available parameters, and the high degree of correlation between parameters [42], we concentrate on four height and complexity parameters. These have shown high discriminatory power between different diet groups in previous studies for both mammals [12,38,40] and non-mammalian vertebrates [29,34,36], and represent key features of the surface topography. Additionally, these parameters stem from different surface texture parameter standards that are commonly employed in DMTA studies: ISO 25178 (*Sq*, *Sdr*), furrows (*metf*) and SSFA (*Asfc*).

1. Height parameters: *Sq* (RMS surface roughness), *metf* (mean depth of furrows).
2. Complexity parameters: *Sdr* (developed interfacial ratio), *Asfc* (area-scale fractal complexity).

Based on these parameters, we also selected location 2 as the focal scanning location from the remaining three scanning locations (2, 4 and 6).

### 2.3. Statistics

Statistical analyses were conducted in JMP Pro v.17.0.0 (SAS Institute Inc., Cary, NC). To test for significant differences between diets, we conducted non-parametric Wilcoxon tests for individuals from each cohort (termination day). If the Wilcoxon test indicated a significant difference, we conducted Dunn's test for joint ranks with a Bonferroni adjustment for multiple comparisons. The same procedure was applied to test for significant differences within diet group and between cohorts (days). Additionally, to test chronological change in DMTA parameters, we conducted Spearman's correlation tests between days and focal DMTA parameters (*Sq*, *metf*, *Sdr*, *Asfc*), respectively for each diet. Principal component analyses were carried out for each day, using 20 DMTA parameters (*Sq*, *Ssk*, *Sku*, *Sp*, *Sv*, *Sz*, *Smc*, *Sdq*, *Sdr*, *Vm*, *Vvv*, *Spd*, *Sk*, *nMotif*, *mea*, *meh*, *matf*, *metf*, *epLsar*, *Asfc*) with a score of >0.5 among the first three principal components, that also showed significant differences between diet groups within one cohort. To facilitate interpretation, we used varimax rotation along the principal axis of components. Descriptive statistics and detailed results of the statistical tests are given in the electronic supplementary material (tables).

## 3. Results

### 3.1. Wear progression

Visual light-microscopic inspection showed almost unworn surfaces for the individuals that received no food for one day after moulting. Only few very fine, shallow scratches were present on the surface (figure 1*a*). Similarly, few or no visible wear marks were found on day 1 for the control pellet and the diets 8sS, 4Ls and 4sVA (figure 2). On the diets containing the largest abrasives, 4lVA and 4lS, cricket mandibles already showed visible wear marks after day 1. From day 3 on, mandibles of crickets on all diets show visible wear. The wear progresses over the course of the experiment, resulting in visually deeper marks in the diets 4Ls, 4lVA, and 4lS. Pellets containing the smallest quartz (8sS) show finer, shallower wear marks. All diets resulted in blunting of mandible teeth, and signs of chipping. Most specimens also showed a deep groove appearing between the first and second mandible tooth at day 21 or 28. The blunt and often broken tooth cusps led to exclusion of scanning locations 1 and 3, while the deep groove covered scanning location 5, which was hence also excluded (figure 1*b*).

### 3.2. Selection of focal scanning location

**Figure 3.**
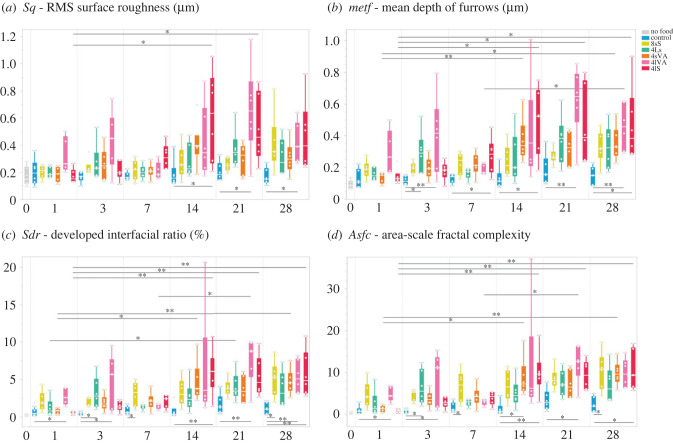
Boxplots of selected DMTA parameters for all diet groups over the course of the experiment. (*a*) *Sq* (RMS surface roughness), (*b*) *metf* (mean depth of furrows), (*c*) *Sdr* (developed interfacial ratio), (*d*) *Asfc* (areal-scale fractal complexity). *p = 0.05, **p = 0.01.

Scanning locations 2, 4 and 6 all showed stable parameter values for the control diet, and low parameter values for individuals that received no food (electronic supplementary material, figure S3). Scanning location 4 showed no significant successive change in the focal height parameter *Sq* (electronic supplementary material, figure S3*b*) and no significant change in parameter values until day 21 for the focal complexity parameter *Sdr*. Overall, parameter values for *Sq* and *Sdr* at location 4 were lower than at location 2, and more similar between diets (compare electronic supplementary material, figure S3). Location 6 showed significantly larger *Sq* and *Sdr* on day 14 than on day 1, but lower values on day 21 and day 28. On day 28, no data from individuals receiving the diet 4lS could be obtained as location 6 was too worn. In contrast, data for location 2 could be obtained on all days. Location 2 showed successive increase in height and complexity parameters for most diets over the course of the experiment, and several significant differences between the control group and diets containing abrasives (figure 3; electronic supplementary material, figure S3). We therefore selected location 2 as a focal position for all analyses.

### 3.3. Height parameters

All diet groups except the control showed a tendency for increasing height parameter values (*Sq*, *metf*) over the course of the experiment (figure 3*a*,*b*). Significant differences between the control and other diet groups were found from day 3 on. For *Sq*, 4lVA showed significantly larger values than the control on days 14 and 21 (figure 3*a*), while 4lS only differed significantly from the control group on day 14. For *metf*, 4lVA showed significantly larger values than the control on day 3, day 21 and day 28 (figure 3*b*), while 4lS showed significantly larger values than the control on days 7, 14 and 28. Among the other diet groups, only 4Ls showed significantly larger *metf* values on one instance for day 3. Crickets fed with 4lVA pellets showed a successive increase of height parameter values (*Sq*, *metf*) from day 1 to day 3 and from day 7 to day 21. In the 4lVA pellet feeders, significantly smaller values for the parameter *metf* were found for day 1 when compared to day 14 and day 28, as well as for day 7 when compared to day 28. Feeding on 4lS pellets resulted in an increase of height parameters until day 14, from where on they plateaued with a high variability. For *Sq*, significantly smaller values within the 4lS group were found when comparing day 1 to day 14 and day 21. For *metf*, day 1 individuals had significantly smaller values than individuals from day 14, day 21 and day 28. The 8sS diet consistently increased for height parameters until day 28, while 4Ls and 4sVA increased from day 1 to day 3, and then from day 7 to day 14 from where they plateaued. The largest height parameter values were reached on day 14 and day 21 in the diet groups 4lVA and 4lS. Besides the diets 4lVA and 4lS, significant increases in the height parameter *metf* over the course of the experiment were found for the diet 4sVA, in which day 1 individuals showed significantly smaller values than day 14 and day 28 individuals. Spearman's correlation tests supported the above findings with significant positive correlations that were found in most diet groups for both *Sq* and *metf*, with the exception of the control group and *Sq* of 4lVA (table 2).

**Table 2.** Results of Spearman's non-parametric correlation tests between days and selected DMTA parameters.

diet	*Sq*		*metf*		*Sdr*		*Asfc*	
	*ρ*	*p*-value	*ρ*	*p*-value	*ρ*	*p*-value	*ρ*	*p*-value
control	**−0.023**	0.892	0.218	0.202	0.354	**0.034**	0.413	**0.012**
8sS	0.457	**0.005**	0.523	**0.001**	0.525	**0.001**	0.531	**0.001**
4Ls	0.423	**0.010**	0.449	**0.006**	0.488	**0.003**	0.457	**0.005**
4sVA	0.381	**0.024**	0.703	**<0.001**	0.730	**<0.001**	0.745	**<0.001**
4lVA	0.261	0.136	0.390	**0.022**	0.409	**0.016**	0.399	**0.019**
4lS	0.645	**<0.001**	0.716	**<0.001**	0.808	**<0.001**	0.833	**<0.001**

### 3.4. Complexity parameters

As seen in height parameters, complexity parameters also showed a tendency for successive increase in all diets except the control over the course of the experiment (figure 3*c*,*d*). On day 7, several diets (4Ls, 4sVA, 4lVA, 4lS) showed lower parameter values than on day 3 but kept increasing again from day 7 to day 21 or day 28. Complexity parameters reached the greatest values for 4lVA on day 21, and for 4lS on day 14, plateauing until day 28. The largest complexity parameter values were seen on day 14 in 4LVA and 4lS, followed by 8sS on day 28. Complexity parameter values were similar between all abrasive-containing diets on day 28. Significant differences between the control group and the 4lVA group were found for *Sdr* on days 1, 3, 21 and 28 (figure 3*c*), and for *Asfc* on days 1, 3 and 21 (figure 3*d*). The control group and 4lS group differed significantly for both *Sdr* and *Asfc* on days 14 and 28. Other significant differences, with the control group showing smaller values for both *Sdr* and *Asfc* were found when comparing to the groups 4Ls (day 3), 8sS (day 7 and 28), and only for *Asfc* in 4sVA (day 14). Between cohorts, the group 4sVA showed significantly smaller values for both *Sdr* and *Asfc* on day 1 than on day 14 and day 28 (figure 3*c*,*d*). For the 4lS group, day 1 had significantly smaller values than individuals on days 14, 21, and 28. The diet group 4lVA only showed significant differences between day 7 and day 21 for both *Sdr* and *Asfc*, while 4Ls had significantly smaller values on day 1 when compared to day 21 only for *Sdr*. Again, the chronological increase of parameter values was supported by Spearman's correlation tests (table 2).

### 3.5. Principal component analysis

**Figure 4.**
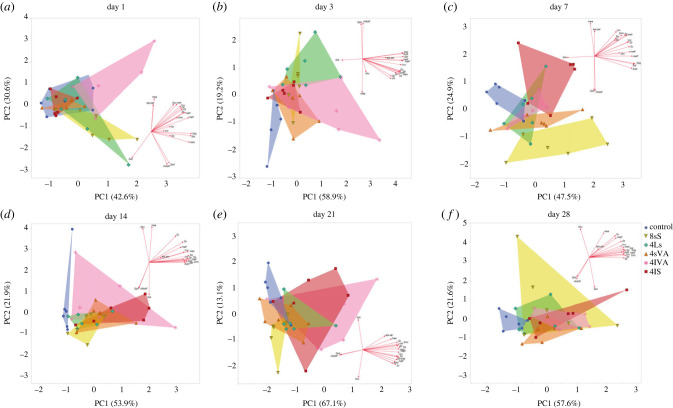
Principal component analysis (PCA) showing PC1 and PC2 for each day. Twenty DMTA parameters were selected according to the best discrimination between diets and PC scores > 0.5 for the first three PCs. (*a*) Day 1, (*b*) day 3, (*c*) day 7, (*d*) day 14, (*e*) day 21, (*f*) day 28.

Principal component analyses were conducted for each day of the experiment separately (figure 4*a*–*f*). On day 1, all diets overlap partially with the space occupied by the control diet. 4lVA, 8sS and 4Ls are showing the strongest differentiation from the control diet and the other diet groups (4sVA, 4lS). On day 3, there is almost no overlap between the control diet and the abrasive-containing diets, which is due to a separation along PC1. 4lVA and 4Ls are most clearly separated from the other diet groups. On day 7, 4lS, 8sS and 4sVA are occupying separate areas in the parameter space and are separated along PC2, while 4Ls and 4lVA overlap. The control group is mostly separated. This separation is maintained on day 14, where the control group is separated along PC1. The abrasive-containing diets show overlap, mainly due to the large variability in the 4lVA group. On day 21, the diet groups 4lVA and 4lS show larger variability. The group 4Ls overlaps with 4lVA and 4lS. Some separation along PC1 is visible, with diet groups containing smaller abrasives (8sS, 4sVA) showing more negative values, while diets with larger abrasives show more positive values (4lVA, 4lS). The control diet is more similar to the diets with small abrasives but separated in PC2. On day 28, all abrasive-containing diet groups are separated from the control along PC1, showing more positive values. 8sS shows the largest variability and overlaps with all other abrasive-containing diets.

### 3.6. Other dental microwear texture analysis parameters

Boxplots for the full set of all 42 computed parameters are included in the electronic supplementary material (figure S2). The corresponding significant differences between diet groups within one cohort, and between cohorts on the same diet are included in the electronic supplementary material (tables).

The control diet showed no significant difference in any parameter between cohorts over the course of the experiment (electronic supplementary material). All other diet groups differed between day 1 and either day 14 (4sVA, 4lS), day 21 (8sS, 4Ls, 4lS) or day 28 (8sS, 4sVA, 4lVA, 4lS) in one or more parameters. Additionally, significant differences were found between day 3 and day 7 (4lS: *new epLsar*), day 3 and day 21 (4lS: *Smr*, *Vm*), day 7 and day 21 (4lVA: *Sdq*, *Sdr*, *metf*, *medf*, *Asfc*), as well as day 7 and day 28 (8sS: *Vm*).

## 4. Discussion

Our controlled feeding experiment with *Gryllus bimaculatus* showed that the mandibles of this insect model show distinct surface wear marks on abrasive-containing experimental diets.

### 4.1. How long does it take until visible wear marks form on insect mandibles on specific diets?

Newly moulted adult crickets showed hardly any wear marks on their mandibles. Only very few and very shallow scratches could be observed (figure 1*a*). These might be due to small defects resulting from the moulting process itself, contact with papers installed as shelters or other individuals immediately after moulting. However, already on day 1 of the experiment, mandibles started to exhibit distinct wear marks. The quality of wear visually differed between the control diet and the abrasive-containing diets from the first day on, with significant differences observed for 8sS and 4lVA.

### 4.2. Does the wear progress over the course of an insect's lifespan?

Not all areas of the mandible showed the same progression of wear. We found that wear as measured by DMTA increased gradually for scanning location 2, a central area on the first mandibular tooth (figure 1*b*). Other locations more proximal on the first (location 6) or second mandibular tooth (location 4) displayed similar wear patterns up to day 14 (electronic supplementary material, figure S3), followed by a sudden increase in surface roughness and complexity for several diets. The observation of progressive mandible wear over insect lifetime is in accordance with previous studies of diverse herbivorous and omnivorous insects [19–23]. As we kept the crickets almost up to the expected end of their adult lifespan of 4–6 weeks, it is to be expected that wear on day 28 has strongly progressed. Crickets were still observed feeding, and we cannot determine whether their feeding efficiency might have been decreased due to mandible wear. However, as differentiation between DMTA patterns on day 28 was weak between diets, we suggest that in this advanced wear stage the wear signatures are less diet-specific than on day 14 or 21, at least on such abrasive-containing diets as employed in this experiment. Natural (plant) diets might differ more subtly in their abrasiveness, thus resulting in overall less distinct wear, and better quantifiable differences at the end of the crickets' lifespan.

### 4.3. Can insect mandible wear be objectively quantified?

We observed a successive increase in visible wear marks, reflected in increasing surface roughness and complexity parameters over the course of the experiment. It is therefore feasible to assume that DMTA is an objective way to quantify progressive wear of the surface of insect mandibles induced by abrasive-containing diets.

### 4.4. Is insect mandible wear diet-specific?

Several abrasive-containing diets resulted in similar parameter values at the end of the experiment (day 28), but the abrasive-free control pellet was significantly different from the diets 8sS, 4lVA and 4lS. Visually, all experimental diets were distinguishable from the abrasive-free control pellet. This highlights that contacts with mechanically challenging mineral abrasives result in more wear of the mandible surface. Our experiment used specifically designed experimental diets that were expected to cause different wear signatures. Even though individuals from the same cohort showed no significant differences between abrasive-containing diets, we found some separation by parameter values, and in the PCAs. Especially days 7, 14 and 21 allowed differentiation between diet groups. Our results show that mandible wear can be diet-specific under experimental conditions, or at least abrasive-specific. The next necessary step is to test whether under more natural conditions, using possible natural diets, mandible wear will show significant, quantifiable differences.

#### 4.5. Universality of the dental microwear texture analysis signal

**Figure 5.**
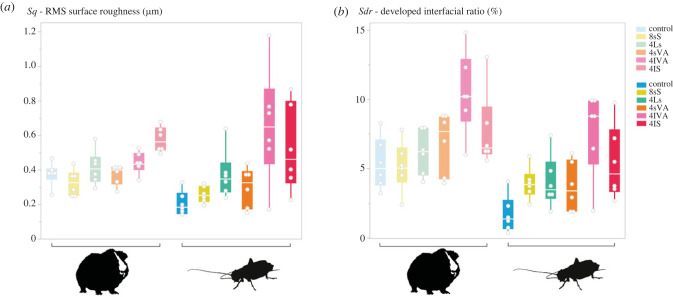
Comparison between cricket DMTA for day 21 and guinea pig DMTA on the same diets measured on the fourth premolar (data from [40]). (*a*) *Sq* (RMS surface roughness), (*b*) *Sdr* (developed interfacial ratio).

The same experimental diets were fed to guinea pigs in a previous feeding experiment [40]. Based on those results, we formed certain presumptions about how mandible wear would differ between diet groups. In guinea pigs, the pellets containing the smallest abrasives (8sS) resulted in smaller lesions, and overall lower surface roughness as expressed by lower height parameters. The largest abrasives (4lVA, 4lS) caused the highest surface roughness, and larger complexity than the other diets. Large volcanic ash (4lVA) showed exceptionally high complexity values. When we directly compare the DMTA patterns for surface roughness (*Sq*) and complexity of the wear pattern (*Sdr*) between guinea pigs from [40] and the crickets from this study, we can see that the spacing between diet groups is comparable. In guinea pigs, the abrasive-free control pellet caused greater roughness and complexity than in the crickets, but the overall pattern of small-abrasive-containing diets (8sS, 4Ls, 4sVA) showing lower surface roughness and complexity than large-abrasive-containing diets (4lVA, 4lS) is consistent between species (figure 5). We have to note that the data for guinea pigs were acquired on a different confocal microscope (µsurf custom) from the data for crickets in this study (VK-9700). Therefore, absolute parameter values are not immediately comparable. In a pilot study, Winkler & Kubo [43] showed that inter-microscope differences exist when scanning the same dataset. But they also showed that even though parameter values differ, relative differences between diet groups are consistently reproduced on different microscopes. Therefore, we assume that the spacing between diet groups in guinea pigs and crickets is consistent, even though data were acquired with different machines. This suggests that tooth and mandible wear by abrasive particles through contacts during feeding is universal, and independent of chewing mechanics, scale, composition and mechanical properties of the feeding apparatus [1,44]. Even though vertebrate teeth and insect mandibles differ in the structure of their food processing tools, both are composed of composite materials with an organic component, hardened by inorganic inclusions [2,45]. A similar observation was already made for the universality of a hard-object feeding signal, which was consistently expressed through high complexity in rats and alligators [34,38].

## 5. Conclusion

Our controlled feeding experiment with *Gryllus bimaculatus*, examining mandible wear through DMTA, supports the abrasive diet-dependent nature of insect mandible microwear. The findings illustrate the temporal aspect of mandible wear on specific abrasive-containing diets. Visible wear marks emerged rapidly, indicating the immediate impact of abrasive-containing diets from as early as day 1. Wear progression varied across mandible locations, showing differential patterns that developed over the experimental period. The progressive nature of wear on a focal location allowed us to quantify diet-induced mandible wear in *G. bimaculatus* through DMTA. The successive increase in mandible wear was reflected in surface roughness and complexity parameters, with abrasive-containing diets significantly differing from the abrasive-free control pellet. While some similarity existed between individuals fed different abrasive-containing diets (with quartz or volcanic ash), differentiation by parameter values and PCA analysis accentuated the diet-specific signatures observable on days 7, 14 and 21.

The comparison between insect and mammalian models (guinea pigs) revealed parallels in mandible and dental wear patterns induced by the same experimental abrasive-containing diets. Despite differences in species, food processing, and body size, the consistent spacing between diet groups implies a universality in tooth and mandible wear caused by abrasive particles during feeding.

As a next step it will be necessary to characterize mandible wear in different insect species and on natural diets to elucidate significant, quantifiable differences in mandible wear under more ecologically relevant conditions. Our research hence can be seen as a framework for future investigations delving deeper into the relationship between diet, mandible wear, and insects and potentially pave the way for using DMTA as a dietary proxy in invertebrates.

## Data Availability

Three-dimensional surface scan data of cricket mandibles are available from the University of Kiel repository: https://doi.org/10.57892/100-34 [46]. Supplementary material is available online [47].
